# Wnt signaling: a promising target for osteoarthritis therapy

**DOI:** 10.1186/s12964-019-0411-x

**Published:** 2019-08-16

**Authors:** Yudan Wang, Xinhao Fan, Lei Xing, Faming Tian

**Affiliations:** 1 Medical Research Center, North China University of Science and Technology, Bohai Road 21, Caofeidian Dis, Tangshan, Hebei 063210 People’s Republic of China; 2 Department of Stomatology, Kailuan General Hospital, Tangshan, Hebei 063000 People’s Republic of China; 3 Department of Geriatrics, Affiliated hospital of North China University of Science and Technology, Jianshe South Road 57, Tangshan, Hebei 063000 People’s Republic of China

**Keywords:** Osteoarthritis, Wnt signaling pathway, Chondrocyte, Osteoblast, Synoviocyte, Osteoclast

## Abstract

Osteoarthritis (OA) is the most common joint disease worldwide and a leading cause of disability. Characterized by degradation of articular cartilage, synovial inflammation, and changes in periarticular and subchondral bone, OA can negatively impact an individual’s physical and mental well-being. Recent studies have reported several critical signaling pathways as key regulators and activators of cellular and molecular processes during OA development. Wnt signaling is one such pathway whose signaling molecules and regulators were shown to be abnormally activated or suppressed. As such, agonists and antagonists of those molecules are potential candidates for OA treatment. Notably, a recent phase I clinical trial (NCT02095548) demonstrated the potential of SM04690, a small-molecule inhibitor of the Wnt signaling pathway, as a disease-modifying oseoarthritis drug (DMOAD). This review summarizes the role and mechanism of Wnt signaling and related molecules in regulating OA progression, with a view to accelerating the translation of such evidence into the development of strategies for OA treatment, particularly with respect to potential applications of molecules targeting the Wnt signaling pathway.

## Background

Osteoarthritis (OA) is a degenerative joint disease typically characterized by articular cartilage degeneration, abnormal bone remodeling with osteophyte formation and subchondral bone sclerosis, and fibrosis and hyperplasia of the synovial membrane [1]. Globally, the World Health Organization estimates that approximately 10% of men and 18% of women aged > 60 years have symptomatic OA, 80% of which suffer from movement limitations [2]. Although various risk factors such as age, obesity, joint trauma, altered biomechanics, and developmental diseases have been recognized, the precise pathogenesis of OA remains unknown. Indeed, a lack of effective treatment strategies for this common chronic condition highlight the fact that the pathological mechanism of OA is far from fully elucidated. During the past few years, treatments and methods for delaying or preventing articular cartilage degeneration have emerged from a greater understanding of the pathogenesis of OA. Accumulating evidence, which has mainly focused on interactions between signaling pathways involved in OA, indicates an important role for Wnt signaling in OA pathogenesis. Therefore, the Wnt signaling pathway is considered a potential target for OA treatment.

Wnt is an extracellularly secreted glycoprotein whose signaling involves 19 Wnt genes and various Wnt receptors that regulate canonical β-catenin-dependent and non-canonical β-catenin-independent signaling pathways. Both downstream pathways are associated with numerous biological processes such as cell proliferation, differentiation, polarization, and fate determination during embryogenesis and late stages of development [3]; as well as the occurrence and development of some diseases – such as increasing evidence for their pathologic role in OA.

## Canonical Wnt signaling pathway

With regard to the canonical β-catenin-dependent Wnt signaling pathway, in the absence of Wnt proteins, β-catenin is degraded n the cytoplasm by the enzyme glycogen synthase kinase 3β (GSK3β) in a “destruction complex” that includes Axin1/Axin2, adenomatous polyposis coli (APC), Dishevelled (Dvl), and casein kinase 1(CK1) in a phosphorylation-dependent manner [4, 5]. However, upon binding of the Wnt signaling molecule to its specific cell membrane receptor, activation of the protein Frizzled (Fzd) and helper receptor low-density lipoprotein receptor-associated protein (LRP5/6) leads to functional signaling. Subsequent activation of Dvl results in dissociation of the multiprotein complex, leading to inactivation of GSK3β. Finally, accumulated β-catenin in the cytoplasm translocates to the nucleus, whereby it interacts with lymphoid enhancer binding factors (LEF) and T-cell factors (TCF) to elicit transcriptional activation of target genes [6].

### Cartilage

Protein levels of Wnt3a and β-catenin were increased, and collagen II was reduced in rat models of normal exercise-induced OA and injured exercise-induced OA groups [7]. Further study demonstrated that activation of β-catenin signaling in specific chondrocytes of adult mice resulted in the development of an OA-like phenotype [8]. Subsequently, transgenic mice with conditional activation of β-catenin signaling in Col2a1- or Agc1-expressing cells were shown to exhibit severe cartilage degeneration, subchondral bone erosion, and osteophyte formation [9]. Similarly, excessive WNT activation following loss of function of the WNT inhibitor Frizzled-related protein FRZB (also called secreted Frizzled-related protein 3, sFRP-3) resulted in increased susceptibility to OA in both humans [10] and mice [11]. In contrast, inhibition of β-catenin signaling in articular chondrocytes resulted in articular cartilage destruction [12], and excessive WNT suppression due to tumor necrosis factor (TNF)-dependent expression of DKK1 in inflammatory arthritis resulted in cartilage and bone destruction [13, 14]. Wnt16-deficient mice developed more severe osteoarthritis with increased chondrocyte apoptosis and reduced expression of lubricin [15], a chondroprotective agent that protects chondrocyte against mechanical damage.

In view of previous studies, moderate WNT activity is essential for chondrocyte proliferation and maintenance of their typical characteristics [16]. However, excessive activity increases chondrocyte hypertrophy and expression of cartilage-degrading matrix metalloproteinases (MMPs) [17], while excessive suppression of Wnt in normal chondrocytes drives OA phenotypes. These findings suggest that a delicate balance of WNT activity is needed for cartilage homeostasis, as both repression and constitutive activation of the β-catenin pathway leads to cartilage breakdown. Additionally, activity of certain mediators and downstream effectors of Wnt/β-catenin signaling are altered in OA.

Wnt1-inducible-signaling pathway protein 1 (WISP1), which positively controls canonical Wnt signaling and aggravates OA pathology [18], is a feature of both experimental and human OA that induces several MMPs, including MMP-3 and MMP-13, as well as the aggrecanases ADAMTS-4 and ADAMTS-5, and is capable of inducing articular cartilage damage in models of OA [17].

Wnt inhibitory factor 1 (Wif-1) blocks Wnt3a-dependent activation of the canonical Wnt signaling pathway in chondrogenic cells [19]. Moreover, WIF-1 expression levels in articular cartilage may be negatively associated with progressive joint damage in patients with OA of the knee [20]. Weng et al. demonstrated an association between upregulated Dickkopf 1 (Dkk1) expression in cartilage and increased OA development, such that intraperitoneal administration of Dkk1 antisense oligonucleotides ameliorated chondrocyte apoptosis and cartilage destruction [21, 22]. Knockout of FrzB, an extracellular antagonist of Wnt signaling, led to enhanced expression of MMPs and accumulation of β-catenin in interleukin 1β (IL-1β)-stimulated chondrocytes, thereby promoting OA development [23]. In addition, catabolic activity in chondrocytes was enhanced by overactivation of the Wnt/β-catenin pathway by sclerostin (SOST) deficiency in several in vitro studies [24–26].

Fibulin-4, an extracellular matrix (ECM) protein reported to be abnormally elevated in human OA chondrocytes [27], augmented the expression of β-catenin and Wnt3a, and diminished GSK3β activation. However, Dkk1 abolished the effect of fibulin-4 on chondrocyte differentiation, suggesting that fibulin-4 activates Wnt/β-catenin signaling and attenuates the expression of ECM [Col2a1, Col10a1, and aggrecan (ACAN)] production and chondrocyte differentiation (Sox6, Sox9, and Runx2) by suppresses the Wnt inhibitor DKK1 [27].

### Synovium

Various members of the Wnt signaling pathway are overexpressed in the synovium during experimental OA [28]. Indeed, increased Wnt signaling (WNT8A and WNT16) in the synovium may potently induce the progression of OA via increased production of MMPs, which are the major protein involved in cartilage destruction [29].

### Subchondral bone

Abnormal remodeling of subchondral bone and osteophyte formation are hallmarks of OA progression [9]. In mice with OA, the canonical Wnt pathway was activated mainly in subchondral bone and forming osteophytes [30]. Knee loading restore subchondral bone remodeling via suppressing abnormal osteoclast activity, by increasing the expression of Wnt3a, and reducing expression of NFATc1 (a master transcription factor for development of osteoclasts), RANKL, TNF-α, and Cathepsin K in a mouse model of knee OA [31]. Low Sirtuin 1 levels in human osteoarthritis subchondral osteoblasts lead to increased SOST expression in mineralization via suppressing Wnt/β-catenin activity [32]. Inhibition of SOST expression play a complicated role in the pathological progression of OA by promoting subchondral bone sclerosis, but potentially inhibiting cartilage proteolysis [33]. Bouaziz et al. showed that SOST-knockout mice with destabilization of medial meniscus (DMM) had high OA scores, with increased expression of aggrecanases and type X collagen [34].

**Fig. 1:**
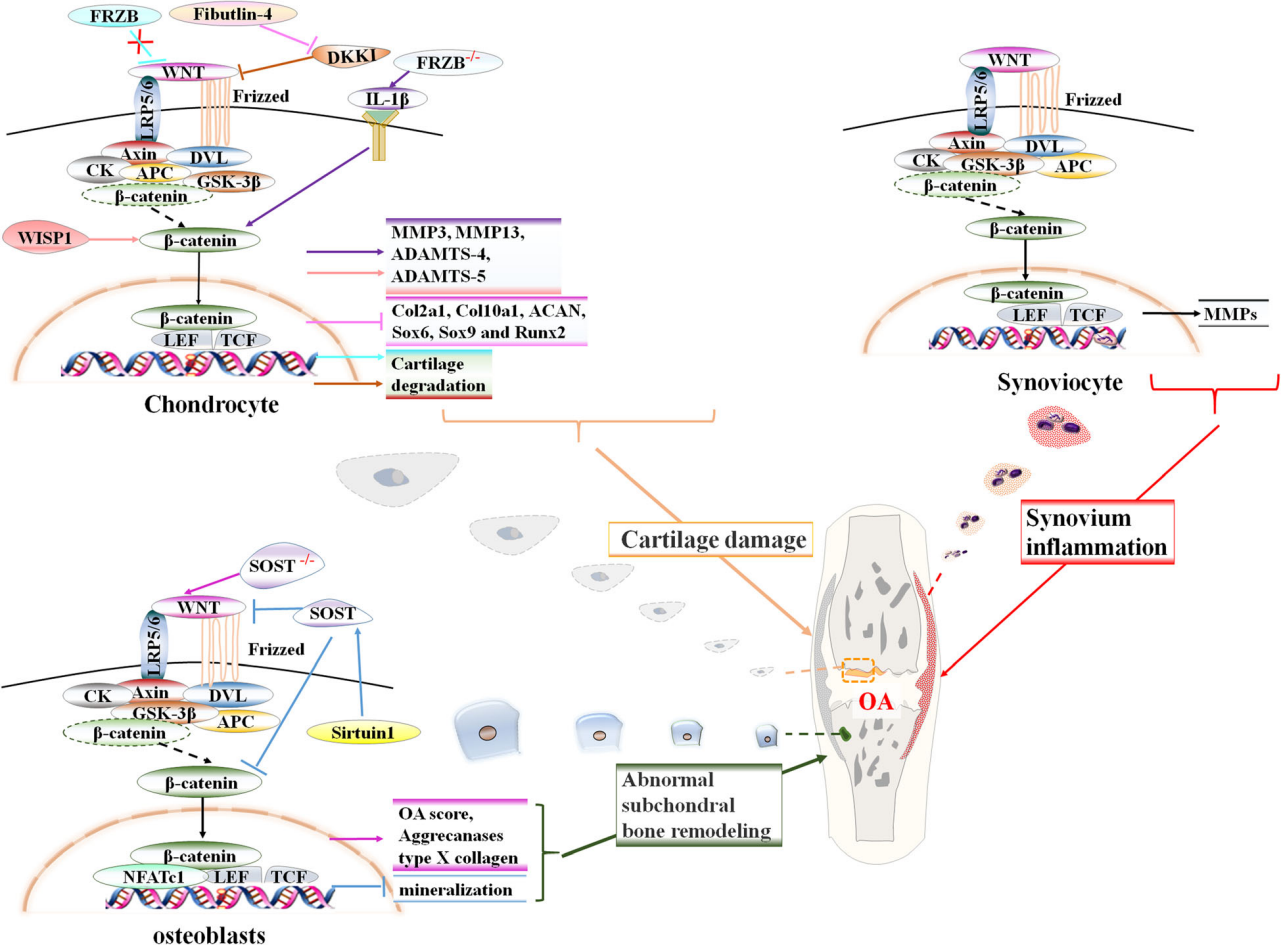
The signaling transduction cascades and cell-specific role of β-catenin-dependent canonical Wnt signaling pathway in regulating chondrocyte, synovial cells and osteoblast metabolism, whereby mediating the process of cartilage degradation, synovium inflammation, as well abnormally activited subchondral bone remodelling in OA development. Events induced by Wnt or β-catenin targeted angonist of antagonist/inhibitor, including DKK1, WISP-1, FRZB, SOST and Fibutlin-4, are marked with event-specific colored line or arrows

Based on the data presented above, we propose that excessively activated canonical Wnt signaling in cartilage, synovium, and subchondral bone plays a critical role in OA development, in both an independent and interacting manner. Accordingly, the Wnt signaling-mediated network that functionally regulates chondrocytes, synovial cells, and osteoblasts/osteoclasts should be highlighted, as the mechanisms underlying these events remain unclear. The degree to which these events share similarities or differ with regard to signaling is not yet fully resolved, nor are interactions between different cell populations (Fig. 1).

## Non-canonical Wnt signaling pathway

Key molecules and cascades in the non-canonical Wnt signaling pathway have been previously summarized [35, 36]. Briefly, non-canonical Wnt signal transduction, which is predominantly activated by Wnt5a, is classified into Wnt/Ca^2+^ and planar cell polarity (PCP) pathways. Through the activation of calcium signaling by phospholipase C/protein kinase C (PKC)/Ca^2+^ and calmodulin-sensitive protein kinase II (CaMKII), the Wnt/Ca^2+^/CaMKII pathway activates the transcription factor nuclear factor associated with T cells (NFAT) to regulate cytoskeletal rearrangements, cell adhesion, and migration. In the PCP pathway, Wnt binds to Fzd receptors, which activates Dvl to trigger Rho/Rho-associated kinase and Rac/c-Jun N-terminal kinase (JNK) signaling, and actin polymerization in stimulated cells. These complex signaling events are integrated to mediate cytoskeletal changes, cell polarization, and motility during gastrulation. Moreover, recent evidence supports the involvement of Wnt5a in inflammatory responses, innate immunity, [37, 38] and particularly, OA development.

### Cartilage

Expressions of Wnt5a in articular cartilage has been positively correlated to progressive damage of knee OA joints [39]. In addition, activation of the Wnt5a/CaMKII pathway correlates with OA development via promoting calcium mobilization and CaMKII phosphorylation in both human and animal models, while CaMKII blockade rescued the loss of chondrocyte phenotype induced by Wnt5a in articular chondrocytes [40]. Similarly, Wnt5A was significantly upregulated in the condylar cartilage of rats in an early temporomandibular joint (TMJ) OA-like model, and was involved in IL-1β-induced MMP expression in TMJ condylar chondrocytes. Activation of Wnt5a expression was facilitated condylar chondrocyte proliferation, hypertrophy and migration though regulated both the expression and transcriptional activity of c-Myc and cyclinD, and thereby inhibited COL2A1, ACAN and promoted MMP13 expression in the condylar cartilage of the rat early TMJ-OA [41]. Blockade of the JNK pathway impaired the effects of Wnt5a on chondrocytes. Yang et al. reported increased expression of Col2a1, a direct transcriptional target of Sox9 (an HMG box transcription factor), in Wnt5a^−/−^ and Col2a1-Wnt5b mice, but decreased expression in Col2a1-Wnt5a transgenic mice; therefore, it is possible that Wnt5a promotes chondrocyte hypertrophy in part by decreasing the transcriptional activity of Sox9 [42].

An in vitro study found that Wnt5a reduced ACAN while promoting MMP1 and MMP13 expression via activating β-catenin independent signaling including JNK and CaMKII in human OA cartilage [43]. Conditioned medium from Wnt5a-expressing cells inhibited type II collagen expression, whereas knockdown of Wnt5a by small-interfering RNA (siRNA) blocked this inhibitory effect; in contrast, Wnt11 promoted type II collagen expression. The opposing effects of Wnt5a and Wnt11 were blocked by inhibitors of JNK and PKC, respectively [26]. These results also indicate different or even opposing roles for Wnt5a in normal conditions and OA progression.

Exosomes, small extracellular microvesicles of endosomal origin, are gaining increasing recognition for their important roles in mediating cell-cell communication [44]. Exosomal mesenchymal stem cell-derived (MSC)-miR-92a-3p-Exos inhibited the progression of early OA and prevented the severe damage to knee articular cartilage via downregulaion of WNT5A expression to promote Sox9 expression and enhanced aggrecan, COMP, and COL2A1 expression in chondrocytes [45].

Taken together, activation of the non-canonical Wnt signaling pathway, predominantly Wnt5a in view of recent data, likely enhances cartilage degradation by stimulating catabolic metabolism of cartilage by upregulating MMP expression and decreasing collagen type II production. Mechanisms underlying this process involve interactions between Wnt5a/CaMKII and key molecules from signaling pathways including JNK, c-Myc, cyclin D1 and Sox9.

### Synovium

With regard to the synovium, Lambert et al. compared the gene expression patterns of synovial cells from inflamed and normal/reactive areas of synovial membrane obtained from the same OA patient; 896 differentially expressed genes were identified, of which Wnt5a and LRP5 were upregulated [46]. In isolated OA fibroblast-like synoviocytes, the combination of TNF-α and IL-17A increased matrix mineralization, alkaline phosphatase (ALP) activity, and expression of Wnt5a, bone morphogenic protein 2 (BMP2), and Runx2, indicating osteogenic differentiation. Wnt5a levels increased upon stimulation with TNF-α alone or in combination with IL-17A [47]. These limited data indicate the liekly involvement of Wnt5a expression in synovial cells in OA development, although further study is needed to reveal precise roles and mechanisms of synovial non-canonical Wnt signaling in OA development.

### Subchondral bone

As the dominant cells in bone remodelling, osteoblasts and osteoclasts are functionally regulated by Wnt5a [48]. Osteoblast lineage cell-specific Wnt5a knockout mice (Wnt5a-cKO) showed low bone quality and reduced bone formation [49]. Similarly, calvarial osteoblast-like cells isolated from Wnt5a^−/−^ mice showed impaired mineralization even treated with BMP2 [50]. These findings indicated that osteoblast-lineage cell-derived Wnt5a is crucial for osteogenisis [50]. Osteoblasts harvested from OA joints exhibited increased expression of non-canonical Wnt5a ligand, ALP activity, and osteocalcin (OC) release compared with normal osteoblasts. Wnt5a stimulated phosphorylation of both JNK and PKC, as well as the activity of both NFAT and activator protein 1(AP-1) transcription factors, also inhibited of Wnt5a expression partially corrected the abnormal mineralization, OC secretion, and ALP activity of OA osteoblasts [51]. Increased *Cxcl12* and *Rankl* gene expression induced by JNK and Ca^2+^/NFAT signaling pathways led to activation of osteoclast differentiation and enhanced subchondral bone turnover [52].

**Fig. 2:**
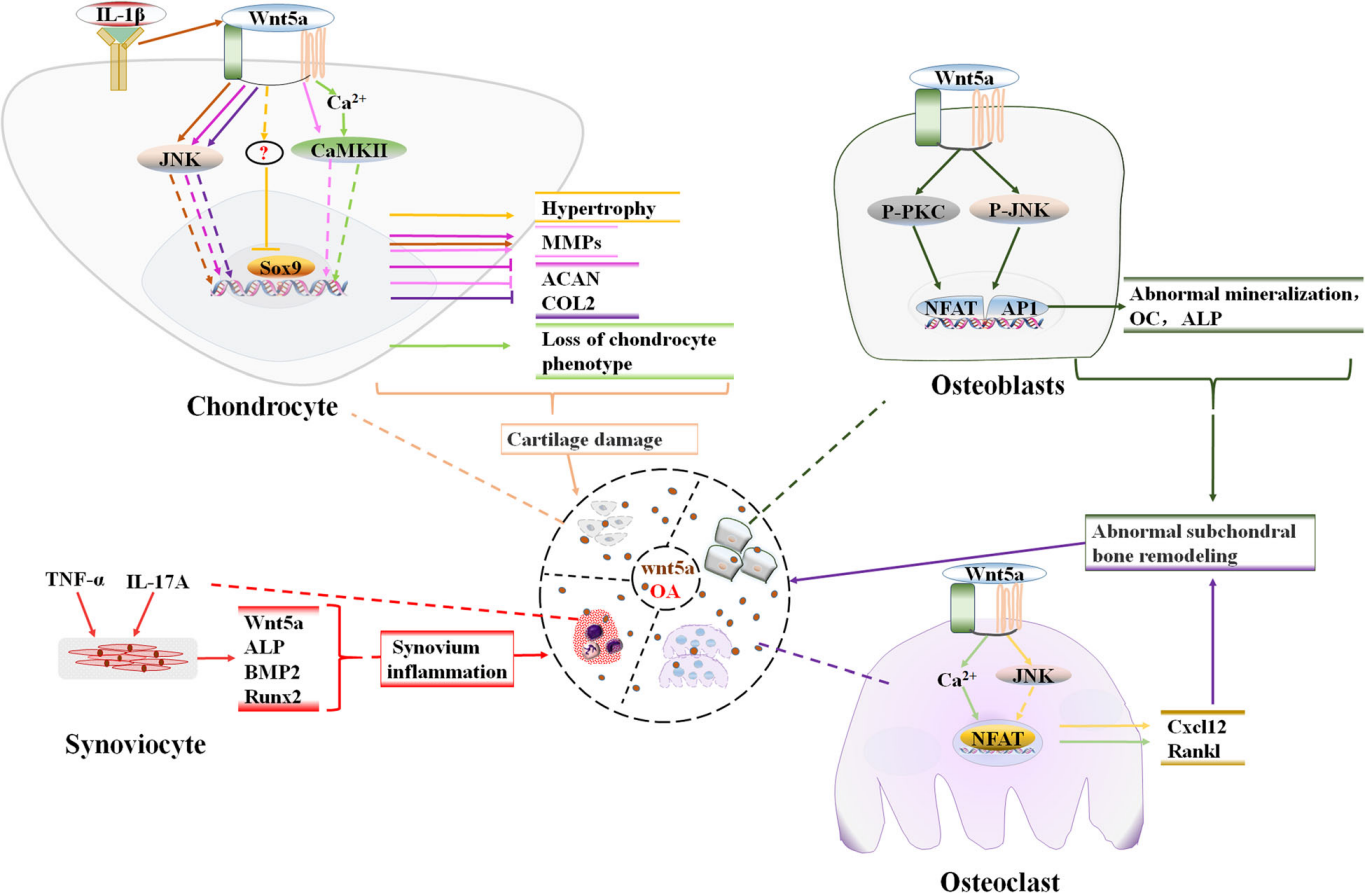
The signaling transduction cascades and cell-specific role of noncanonical Wnt signaling, predominantly activated by wnt5a, in regulating chondrocyte, synovial cells, osteoblast and osteoclast metabolism, including mediated IL-1 beta stimulated chondrocyte catabolism, TNF-alpha and IL-17A induced synovium inflammation, as well abnormal mineralization of osteoblast and osteoclast differentiation, all these process are promised to be involved in OA development

Collectively, these data indicate Wnt5a signaling and its downstream cascades may lead to an abnormal balance between osteoblasts and osteoclasts, which can increase subchondral bone remodeling and participate in excessive mineralization or even osteophyte formation. Cell-specific modulation of Wnt5a expression and its receptor in osteoblasts or osteoclasts would provide direct evidence to elucidate the precise role of this signaling pathway in OA (Fig. 2).

## Proposed Wnt signaling-mediated network in OA development

**Fig. 3:**
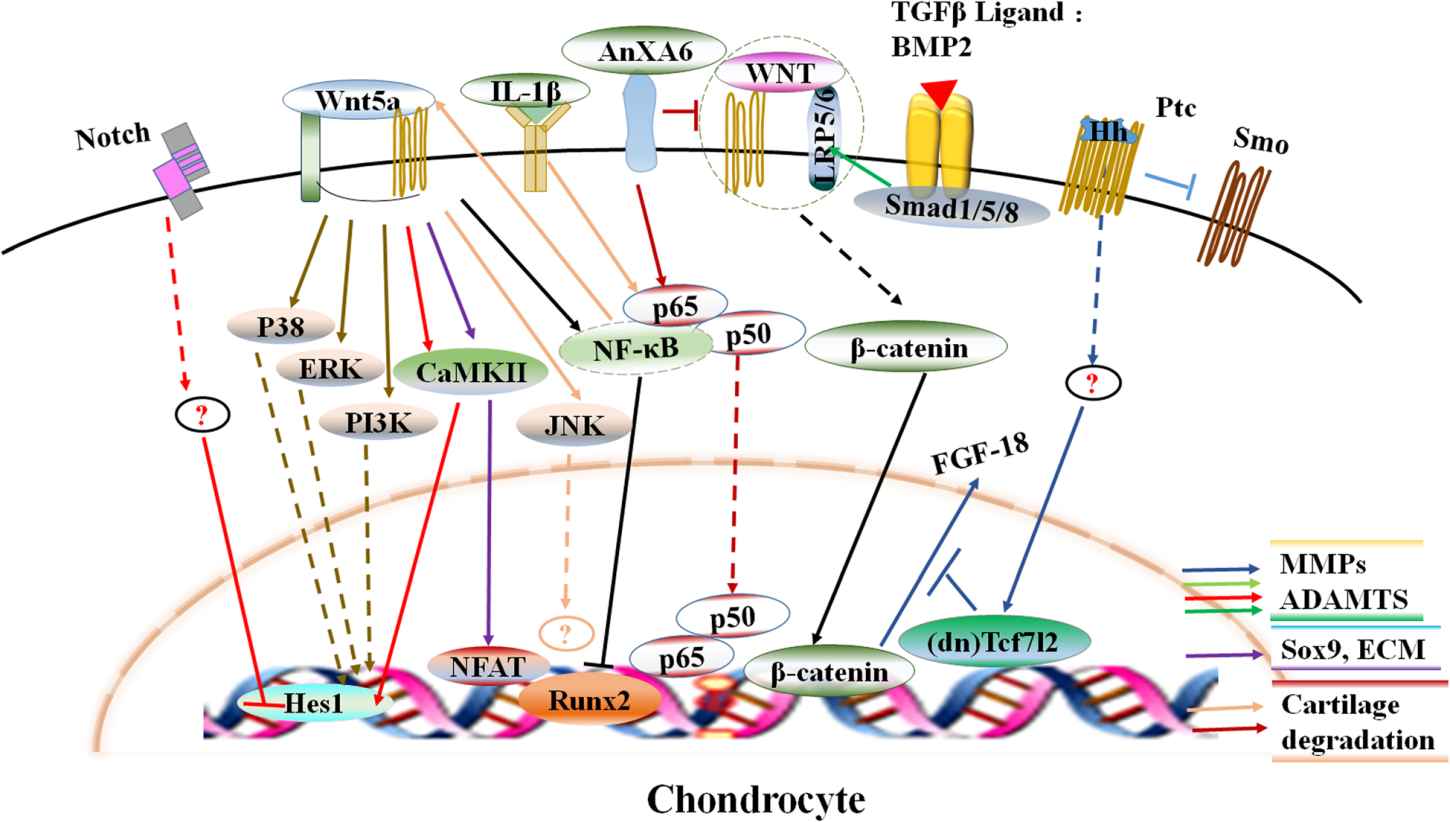
Proposed model of the role of canonical and noncanonical wnt signaling pathway mediated network that regulating chondrocyte function, the activiation of Wnt signaling and their interaction between either canonical and noncanonical, or Hedgehog, MAPK, NF/κB, BMP/TGF-β/Smad and Notch signaling pathways, are assiciated with chondrocyte differentiation, hypertrophy, catabolism, anabolism and thereby involed in OA development, and marked with event-specific colored line or arrows

**Fig. 4:**
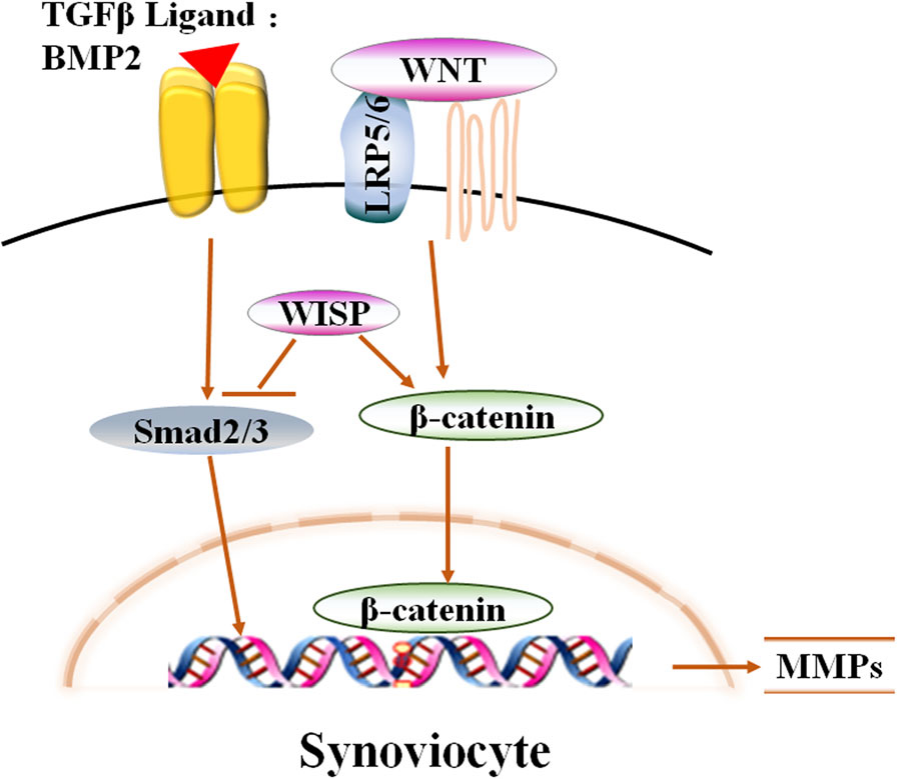
WISP stimulates MMPs expression in synovium and cartilage through inhibiting TGF-b /Smad 2/3 signaling and activating the accumulation of b-catenin, which serves to enhance the synovium inflammation

**Fig. 5:**
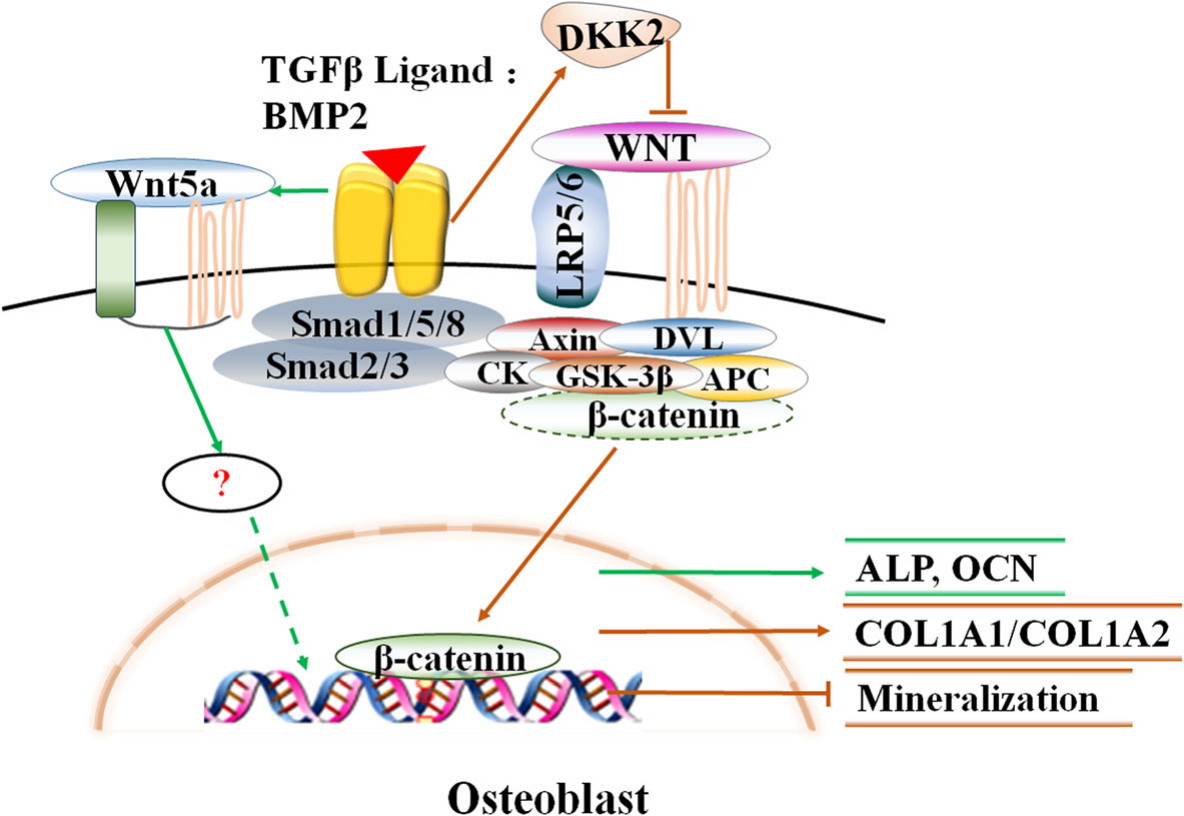
Interaction within TGF-b/BMP, canonical and non-canonical signaling pathway in osteoblast, in addition to induces DKK2 to inhibit the canonical Wnt signaling pathway, TGF-b/BMP signaling also up-regulates the expression of Wnt5a In osteoblasts, and subsequently stimulating the expression of a number of genes involved with osteogenesis

With respect to the mechanisms by which the Wnt signaling pathway participates in OA development, a series in-depth studies indicated that the pathogenesis of OA is, at least in part, the result of interactions between Wnt and multiple signaling pathways. In normal chondrocytes, the network formed by signaling pathways such as Wnt, BMP, Hedgehog, etc. are needed to maintain their normal phenotype [53]. Based on recent literature, signaling pathways including Wnt, BMP/transforming growth factor β (TGF-β), parathyroid hormone (PTH), Hedgehog, Notch, hypoxia-inducible factor (HIF) and Hippo signaling exhibit abnormal activity [54] and interactions with each other. Thus, this network is a crucial participant in OA development (Figs. 3, 4 and 5)

### Nuclear factor-kappa B (NF-κB)

NF-κB signaling is important for several biological and pathological processes [55], such as embryonic immunity, apoptosis, angiogenesis, development, and proliferation [56]. Kirsch et al. showed that overexpression of annexin A6 (a member of the highly conserved annexin family of Ca^2+^-dependent membrane-binding proteins) interacted with p65 resulted in increased nuclear translocation and retention of the active p50/p65 NF-kB complex, whereas plasma membrane-associated AnxA6 interfered with the membrane-association of the Wnt signalosome complex required for the activation of Wnt/β-catenin signaling in human cartilage degradation during OA pathology [57]. Similarly, IL-1β stimulated Wnt5a expression through activation of NF- κB and the subsequently overexpression of p65 in chondrocytes, while BAY11–7082, a specific inhibitor of IκBα-phosphorylation, abrogated the induction of Wnt5a by IL-1β in the cartilage destruction caused by arthritis [58]. In addition, Wnt5a acts to increase chondrocyte differentiation at an early stage through CaMKII/NFAT-dependent induction of Sox9; in contrast, Wnt5a represses chondrocyte hypertrophy via NF-κB-dependent inhibition of Runx2 expression [59]. This indicates a dual role of Wnt5a to promote early chondrocyte differentiation in a stage-dependent manner while repressing chondrocyte hypertrophy [59]. Thus, signaling factors may regulate chondrocytes hypertrophy and degeneration via interactions between Wnt and NF-κB signaling pathways.

### Bmp/TGF-β

Crosstalk between Wnt and BMP pathways not only participates in chondrocyte hypertrophy and matrix degradation, but also stimulates bone formation via chondrogenic or osteogenic differentiation. In vitro, BMP2-induced Wnt/β-catenin signaling pathway activation through increased β-catenin nuclear translocation and LRP-5 expression and that the BMP-2-induced LRP-5 upregulation is mediated through Smad1/5/8 binding on LRP-5 promoter, resulted in MMPs and ADAMTS-5 expression, and hypertrophic maturation of chondrocytes by stimulating collagen X expression [60]. In addition, gradual increases in WNT and BMP signaling in joints with increasing age may contribute to the increased incidence of OA development in older patients with joint defects [61].

Nemoto et al. demonstrated an association between BMP2-mediated osteoblastic differentiation and increased Wnt5a and Ror2 expression in vivo and in vitro, and silencing gene expression of Wnt5a and Ror2 resulted in suppression of BMP2-induced expression of ALP and osteocalcin (OCN), suggesting that Wnt5a and Ror2 signaling form a substantial component of BMP2-mediated osteoblastic differentiation in a Smad independent pathway [62]. Compared with normal osteoblasts, OA osteoblasts dependent on TGF-β1 expression of DKK2 (the antagonists of Wnt signaling) increased and decreased Wnt/β-catenin signal transduction, leads to an increase of the COL1A1 to COL1A2 ratio, as well as to an reduce in mineralization following BMP-2 stimulation [63].

Previous studies suggest that the TGF-β/Smad pathway plays a critical role in regulation of articular chondrocyte hypertrophy and maturation during OA development [64–66]. Overexpression of WISP1 in the synovium and cartilage leads to increase in MMPs, as is found in OA conditions, may further aggravate OA pathology by decreasing TGF-β (Smad2/3) signaling and via a positive feedback mechanism on canonical Wnt signaling [67]. Crosstalk between β-catenin and TGF-β was reported in hypertrophic regulation of mesenchymal stem cells (MSCs) [68]. Continuous co-activation of these two signaling pathways during chondrogenesis of MSCs resulted in increased secretion of PTH-related peptide (PTHrP) and expression of cyclin D1, which may have a role in the inhibition of chondrocyte hypertrophy by suppressed expression of collagen type X, RUNX2, and alkaline phosphatase [68]. Indeed, interactions between TGF-β and β­catenin-dependent Wnt signaling, as well as the balance between these two pathways, may play a vital role in regulating both OA development and maintenance.

### PTH

Interestingly, Wnt/β-catenin signaling regulates initiation of chondrocyte hypertrophy by antagonizing PTHrP signaling, whereas it acts independently of PTHrP signaling to control the final maturation of hypertrophic chondrocytes [69]. Ma et al. demonstrated that PTH (1–34) increased mRNA expression and protein levels of PTH1R and β-catenin by repressing SOST and Dkk1 expression, and reduced both Mankin scores and Runx2 expression in an anterior cruciate ligament transection with DMM rat OA model [70].

### Hedgehog

Rockel et al. reported that in adult chondrocytes, activated hedgehog signal induction expression of dominant negative equivalent TCF7L2 (dnTCF7L2) isoforms, and that increased expression of TCF7L2 protein isoforms limited signaling by β-catenin, resulting in an inhibition of expression of FGF18, leading to cartilage degeneration via induction of expression ADAMTS4 and MMP13, which are involved in cartilage degeneration as part of OA [71]. Therefore, the balance Hedgehog and β-catenin signaling is critical for maintenance of articular cartilage in adult mouse model of OA.

### Notch

Notch is actively involved in various life processes including osteogenesis, and that Hes1, an essential mediator of Notch signaling, generally mediates Notch signaling by repressing expression of target genes [72, 73]. CaMKII causes Hes1 to switch from a transcriptional repressor to transcriptional activator, thereby enhancing the expression of catabolic factors such as Adamts5, Mmp13, IL-6, and IL-1 receptor-like 1 in articular cartilage to promote OA development [74]. Therefore, interactions between Notch signaling and CaMK2 in non-canonical Wnt signaling pathways are predicted to be involved in OA development.

### Hypoxia-inducible factor 1α (HIF1α)

HIF1α is a crucial hypoxic factor for chondrocyte growth and survival during development [75]. HIF1α inhibits β-catenin signaling by blocking transcription factor 4 (TCF4) β-catenin interaction and down-regulates MMP13 expression, thereby alleviating cartilage lesions, whereas the TCF4 β-catenin signaling induces an OA phenotype in mice [75]. Furthermore, ΔHif1α ^chon^ mice with OA that were intra-articularly injected with PKF118–310, an inhibitor of the TCF4/β-catenin interaction, exhibited reduced cartilage degradation and MMP13 expression [75].

### Hippo/YAP

The Hippo/YAP signaling pathway is important for mediating organ size and tissue homeostasis, and inhibition of YAP using YAP siRNA is a promising way to prevent cartilage degration in OA [76]. Wnt5a and Wnt5b (highly expressed in both synovial mesenchymal stem cells and exosomes) transported by exosomes activates YAP via suppression of the Wnt signaling pathway target gene SOX9 expression and ECM secretion to enhance proliferation and migration of chondrocytes, which was overcome by overexpressing miR-140-5p in SMSCs and using the SMSC-140-Exos [77].

### Other

An in vitro study found that Wnt5a reduced ACAN while promoting MMP1 and MMP13 expression via activated β-catenin independent signaling including p38, extracellular signal-regulated kinase (ERK) and phosphoinositide 3-kinase (PI3K) in human OA cartilage [43].

## Therapy for osteoarthritis

Management of the pathogenesis of OA has become central for treatment and alleviation of related clinical symptoms. Osteoarthritis Research Society International recommendations cover the use of non-pharmacological (e.g. water-based exercises, electrical nerve stimulation), pharmacological modalities (e.g. acetaminophen, non-steroidal anti-inflammatories, intra-articular injection of corticosteroids) and surgical modalities (e.g. total joint replacement, unicompartmental knee replacement) [78]. Although traditional pharmacological therapies are effective for relieving pain, they are incapable of reversing cartilage damage and frequently associated with adverse events [79]. Additionally, the risk of implant revision associated with age is a potential lifetime risk and financial burden for patients undergoing hip or knee joint replacement, especially for men aged 50–55 years [80].

In recent years, pluripotent stem cells and regenerative medicine strategies have been considered promising to repair cartilage damage in OA. However, their long-term effects remain uncertain based on limited clinical data. In view of the important role of Wnt signaling pathways and cascades molecules play in OA, they might be potential target for the treatment of OA.

### Therapy targeting or acting via Wnt signaling pathways

Here we summarize emerging therapies that target or act via Wnt signaling pathways, in terms of small molecule antagonists or agonists, herbs, enzymes, and tissue engineering and so on.

### Cartilage

#### Small-molecule inhibitors

First and most importantly, SM04690 was shown to elicit protective effects on cartilage during joint destruction in a preclinical model of knee OA [81]. A further phase II clinical trial of SM04690 (Samumed) for intra-articular therapy of moderate-to-severe knee OA showed that it improved cartilage degradation without toxicity [82, 83]. Two small molecule inhibitors, the stapled peptides StAx-35R (stapled β-catenin binding domain of Axin) and SAH-Bcl9 (stapled peptide derived from the Bcl9 homology domain-2) have been established to inhibit β-catenin transcriptional activity [84, 85]. More recent research suggests that SAH-Bcl9 and StAx-35R inhibited chondrocyte phenotypic shifting of preserved human OA cartilage explants, resulting in increased *SOX9* and *ACAN* gene expression, and decreased COL10A1 expression [86].

LRP5 plays an essential role in Wnt/β-catenin signaling mediated OA cartilage destruction by upregulating catabolic factors (for example, MMP3 and MMP13) and downregulating the anabolic factor type II collagen [87]. These effects were ameliorated in Lrp5-knockdown mice, which exhibited reduced cartilage destruction [87].

Lorecivivint inhibited CDC-like kinase 2-mediated (CLK2) phosphorylation of 61 serine/arginine-rich (SR) proteins and DYRK1A-mediated (dual-specificity tyrosine phosphorylation-regulated kinase 1A) phosphorylation of SIRT1 and FOXO1, suggested a novel mechanism for Wnt pathway inhibition, enhancing chondrogenesis, inhibited expression of cartilage catabolic enzymes, and anti-inflammation [88]. It is a safe and well tolerated treatment for OA in vivo and clinical trials. (NCT02095548, NCT02536833, NCT03122860).

#### Herb

Psoralen, the active ingredient of Fructus Psoraleae (dried ripe fruit of *Psoralea corylifolia* L.), reportedly promotes chondrocyte proliferation by activating the Wnt/β-catenin signaling pathway, and may play an important role in OA treatment [89]. Additionally, tetrandrine [90] and berberine [91] were shown to exert protective effects on OA chondrocytes.

Artemisinin (ART) inhibition of OA progression and cartilage degradation through upregulation of FRZB in IL-1β-induce chondrocytes and downregulation of the expression of β-catenin, which suggests that it may act as a Wnt/β-catenin antagonist to reduce the release of inflammatory mediators and enhance cell proliferation, glycosaminoglycan deposition, and prevent cartilage apoptosis and degeneration [92].

#### Enzyme

Ma et al. reported that knockdown of peroxiredoxin 5 (Prdx5) by RNA interference activated apoptosis of OA chondrocytes, which was mediated through decreased scavenging of endogenous reactive oxygen species and promotes the nuclear translocation of β-catenin, decreases GSK-3β activity, and enhances β-catenin/TCF-dependent transcription in osteoarthritic chondrocytes [93]. Therefore, Prdx5 may play a protective role in human OA cartilage degeneration.

#### Engineering cartilage

Kim et al. reported that the tri-butanoylated N-acetyl-D-galactosamine analog (3,4,6-O-Bu3GalNAc), a multifunctional carbohydrate-based drug candidate, improved cartilage tissue production in 3D cultures in vitro by inhibiting Wnt/β-catenin signaling [94], suggesting that this unconventional carbohydrate-based drug may prevent OA progression and limit inflammation in OA.

Praxenthaler et al. shows a correlation of WNT/β-catenin activity with de- and re-differentiation and ECM deposition in human articular chondrocytes with time in 3D culture, thus establishing that the mechanical loading response of chondrocytes is modulated by WNT/β-catenin activity levels [95]. Therefore, the balance between Wnt signaling, mechanical load sensors, and ECM signaling is important for cartilage maintenance and breakdown.

#### Antidepressant

Mianserin suppresses R-spondin 2-induced activation of Wnt/β- catenin signaling in chondrocytes and prevents cartilage degradation in a rat model of osteoarthritis [96]. Fluoxetine, an antidepressant in the class of selective serotonin reuptake inhibitors (SSRI), decreased expressions of Axin2 and MMP13, suppressed degradation of proteoglycans, and increased expression of Sox9 in chondrogenically differentiated ATDC5 cells [97]. Thus, intraarticular injection of fluoxetine may improve OA progression and inhibited the accumulation of β-catenin in rats OA model.

#### MircoRNA

MiR-320c suppress the Wnt/β-catenin signaling pathway through the downregulation of β-catenin protein level in nucleus, inhibiting chondrogenic degeneration in osteoarthritis [98]. Overexpression of miR-138 promotes chondrocytes proliferation while it inhibits apoptosis in OA through the WNT/β-catenin signaling pathway via downregulation of NIMA-related kinase 2 (NEK2) [99].

### Synovial

After stimulation with IL 1β or fibronectin fragments, and blockage of Wnt signaling by DKK1, Selene et al. showed that ERK inhibition decreased Runx2 activation, ADAMTS 7, 12 expression and cartilage oligomeric matrix protein (COMP) degradation in OA synovial fibroblasts (SF) [100].

### Subchondral bone

Osteoclast activity plays a significant role in the interaction between articular cartilage and subchondral bone, and knee loading may suppress osteoclastogenesis through the Wnt signaling pathway to serve as a treatment for OA mice [31]. Therefore, knee loading-induced inhibition of osteoclast activity may be a new noninvasive OA treatment strategy.

Burt et al. reported that a fibroblast growth factor 23 (FGF23) neutralizing antibody was able to partly ameliorate OA abnormalities in subchondral bone and reduce degradative/hypertrophic chondrogenic marker expression in high-molecular-weight joints in vivo [101]. Moreover, they identified FGF23-mediated Wnt/β-catenin signaling as a candidate pathway for the treatment or prevention of OA [101].

#### Physiotherapy

Pulsed electromagnetic field (PEMF) treatment prevented subchondral trabecular bone loss and preserved subchondral trabecular microarchitecture in a low-dose monoiodoacetate rat model, at least partially via Wnt/β-catenin signaling [102]. In addition, extracorporeal shock wave treatment (ESWT) improved symptoms, inhibited cartilage degeneration, and promoted rebuilding of subchondral bone in OA rats, the mechanism of which involved activation Wnt5a/Ca^2+^ signaling in bone marrow-derived MSCs [103].

### Dual-targeting of cartilage and subchondral bone

Dkk1 competitively binds to Wnt co-receptors LRP5/6 to inhibit Wnt signal transduction [104, 105]. Serum levels of Dkk1 predict the progression of hip OA [106] and are inversely correlated with the severity of knee OA [107]. Hwanhee et al. reported that cartilage-specific overexpression of Dkk1 exerts a protective effect against OA cartilage destruction by inhibiting the canonical Wnt pathway [108]. Dkk1-mediated control of Wnt/β-catenin activation in subchondral bone leads to reduced severity of OA [30]. Dkk1 can decrease OA progression, likely by reducing the severity of osteophytes [30]. Interestingly, by inhibiting Dkk1, Diarra et al. were able to reverse the bone-destructive pattern of a mouse model of rheumatoid arthritis to the bone-forming pattern of OA [13]. In this manner, no overall bone erosion resulted, although bony nodules, so-called osteophytes, did form [13].

### Therapies mediated by interactions with other signaling pathways

The pathogenesis of OA is the result of multiple interacting factors. Although the mechanism is unclear, Wnt signaling has been shown to contribute to whole joint disease. Recent studies have demonstrated interactions between multiple signaling pathways in OA, including canonical and non-canonical Wnt, Hedgehog, TGF-β, and NF-ĸB signaling pathways, which represent potential targets for the treatment of OA.

#### Palmatine

Studies have shown that palmatine (a member of the protoberberine class of isoquinoline alkaloids) may delay the progression of OA, as determined by assessments of articular cartilage in a rabbit OA model and cultured rabbit chondrocytes stimulated with IL-1β [109, 110], possibly via Wnt/β-catenin and Hedgehog signaling pathways [111].

#### Dickkopf 3 (DKK3)

Snelling et al. provided evidence that Dkk3, a non-canonical member of the Dkk family of Wnt antagonists, was upregulated in OA, whereby it mediated protective effects on cartilage partially through upregulation of TGF-β signaling and inhibition of Wnt signaling [112]. Thus, Dkk3 treatment may prevent OA-induced cartilage degeneration, and early intervention targeting Dkk3 may slow the progression of OA.

#### Specnvezhenide (SPN) and licochalcone a

SPN is an extracted agent of the fruit of *Ligustrum lucidum* [113]. Ma et al. demonstrated that IL-1β-induced transcriptional activity of NF-κB and Wnt/β-catenin pathways was greatly decreased by treatment with SPN, which protected cartilage in vitro by decreasing protein levels of MMPs and inflammatory factors, and increasing levels of collagen II and Sox9 [113]. Similarly, Chen et al. demonstrated that licochalcone A can inhibit IL-1β-induced catabolism via NF-κB and Wnt/β-catenin signaling pathways in rat chondrocytes [114].

The anti-osteoarthritic effects of emodin by inhibiting the expression of MMPS and ADAMTS via the suppression of IκB-α degradation, the down-regulation of IKK-β and NF-κB p65 levels, as well the reduction of Wnt/β-catenin activity by inhibiting β-catenin in-vitro and in-vivo [115]. In vivo, cartilage treated with Costunolide showed attenuated degeneration and lower mankin scores compared to the OA group [116]. Costunolide inhibits p65 phosphorylation and the transfer into the nucleus, and inhibits the Wnt signaling pathway through the activation level of β-catenin and the transfer of β-catenin into the nucleus induced by IL-1β in rat chondrocytes [116].

#### Cationic amphipathic peptide

Hou et al. reported that the cationic amphipathic peptide designated as p5RHH is capable of siRNA transfection without significant cytotoxicity at all tested doses [117]. Yan et al. subsequently showed that p5RHH NF-κB siRNA nanotherapy mediates its chondroprotective effect partially by maintaining cartilage autophagy/homeostasis via modulation of AMP-activated protein kinase, mechanistic target of rapamycin C1, and Wnt/β-catenin activity [118].

## Conclusions and perspectives

Both canonical and non-canonical Wnt signaling pathways are excessively activated during OA development. A series of studies have revealed that cell-specific modulation of Wnt or its downstream effectors can lead to or prevent aberrant changes in OA tissues. The underlying molecular mechanisms involve other signaling pathways such as Hedgehog, NF/κB, BMP/TGF-β/Smad, PTH, Notch and HIF1α, − more specifically, the network they construct. Further studies using various knockdown or knock-in strategies to test functional roles of these components are necessary to better understand the nature of these events. Although the details of cell-specific molecular events, especially the cascades by which Wnt signaling interacts with the other signaling pathways described above, remain to be elucidated, key points of the network composed by these interacting pathways are promising potential targets for OA treatment. Notably, a recent phase I clinical trial (NCT02095548) demonstrated that the Wnt signaling pathway inhibitor SM04690 has potential as a DMOAD The development of additional emerging genetic therapies or small molecules targeting the Wnt signaling pathway, though only currently supported by evidence from animal studies, should be encouraged. Moreover, in vivo experiments and clinical data are needed to clarify mechanisms of action and clinical value of the signaling networks affected by natural antioxidants Prdx5 and Traditional Chinese Medicines containing ART and psoralen, as they have been shown to delay the process of OA via Wnt signaling. In addition, we should develop a more in-depth understanding of the beneficial mechanisms of PEMF and ESWT physiotherapy to provide more powerful evidence for clinical treatment of OA. Moreover, new therapeutic strategies such as engineered cartilage, pluripotent stem cells, and regenerative technologies should be explored to seek more effective therapies for OA. However, before seeking new agents for OA treatment that target the Wnt signaling pathway, further study is needed to test the efficiency and safety of SM04690 and its derivates before they are prescribed as agents for OA treatment.

## Data Availability

Not applicable.

## Abbreviations

ACAN: Aggrecan
ALP: Alkaline phosphatase
AP-1: Activator protein 1
APC: Adenomatous polyposis coli
ART: Artemisinin
BMP2: Bone morphogenic protein 2
CaMK II: Calmodulin-sensitive protein kinase II
CK1: Casein kinase
CLK2: CDC-like kinase 2
COMP: Cartilage oligomeric matrix protein
Dkk1: Dickkopf 1
DMM: Destabilization of medial meniscus
DMOAD: Disease-modifying oseoarthritis drug
Dvl: Dishevelled
DYRK1A: Dual-specificity tyrosine phosphorylation-regulated kinase 1A
ECM: Extracellular matrix
ERK: Extracellular signal-regulated kinase
ESWT: Extracorporeal shock wave treatment
FGF23: Fibroblast growth factor 23
FRZB/SFRP-3: Frizzled-related protein 3
Fzd: Frizzled
GSK3β: Glycogen synthase kinase 3β
HIF: Hypoxia-inducible factor
IL-1β: Interleukin 1β
JNK: c-Jun N-terminal kinase
LEF: Lymphoid enhancer binding factors
LRP5/6: Low-density lipoprotein receptor-associated protein
MIRRNA: MircoRNA
MMPs: Matrix metalloproteinases
MSC: Mesenchymal stem cell
MSCs: Mesenchymal stem cells
NEK2: NIMA-related kinase 2
NFAT: Nuclear factor associated with T cells
NF-ΚB: Nuclear factor-kappa B
OA: Osteoarthritis
OCN: Osteocalcin
PCP: Planar cell polarity
PEMF: Pulsed electromagnetic field
PI3K: Phosphoinositide 3-kinase
PKC: Protein kinase C
Prdx5: Peroxiredoxin 5
PTH: Parathyroid hormone
PTHrP: PTH-related peptide
SF: Synovial fibroblasts
SiRNA: Small-interfering RNA
SOST: Sclerostin
SPN: Specnvezhenide
SR: Serine/arginine-rich
SSRI: Selective serotonin reuptake inhibitors
TCF: T-cell factors
TGF-β: Transforming growth factor β
TMJ: Temporomandibular joint
TNF: Tumor necrosis factor
Wif-1: Wnt inhibitory factor 1
WISP1: Wnt1-inducible-signaling pathway protein 1
